# Supplementary material for: Liposomal Encapsulation of Carob (Ceratonia siliqua L.) Pulp Extract: Design, Characterization, and Controlled Release Assessment
Source: Pharmaceutics. 2025 Jun 13;17(6):776. doi: 10.3390/pharmaceutics17060776 (PMC12196410; doi:10.3390/pharmaceutics17060776)
Supplement: Supplementary file 1 [file pharmaceutics-17-00776-s001.zip › pharmaceutics-3618022-supplementary.pdf]

Results obtained from the release studies of carob extract and carob extract-loaded liposomes were analyzed to determine the diffusion coefficients (D) and diffusion resistances (R) derived from liposomes in simulated gastric and intestinal fluids. The diffusion of polyphenols from liposomes to the receptor fluid through the membrane can be approximated using Fick's second law, shown in Equation (1):

$$\ln\left(\frac{C_d^0 - C_r^0}{C_d - C_r}\right) = D\,\beta\,t \quad (1)$$

where C_d and C_r are the concentrations of carob polyphenols detected in the donor and receptor compartments at time t; C_d^0 and C_r^0 are the concentrations of carob polyphenols at the beginning of the study; and D is the diffusion coefficient. The geometrical constant β value, typical for the Franz cell geometry, was 2.49×10^4 m^{-2}.

The diffusion coefficients of carob polyphenols from liposome dispersion were calculated from the slope of the linear part of a curve defined by plotting $\ln\left(\frac{C_d^0 - C_r^0}{C_d - C_r}\right)$ *vs.* time.

The overall diffusion resistance, R, was calculated using Equation (2):

$$R = \frac{\delta}{D} \quad (2)$$

where δ is the membrane thickness.

Diffusion resistance represents the cumulative resistance of a semipermeable acetate cellulose membrane and the resistance of a liposomal bilayer. The contribution of the resistance, which is generated by the synthetic membrane, was determined from the diffusion of polyphenols from the pure carob extract. Then, the liposome resistance was determined by subtracting the synthetic membrane resistance from the overall diffusion resistance.

Table S1. Row data on viscometry and tensiometry measurements of carob extract-loaded liposomes.

Day	Liposomes	Viscosity (mPa•s)	Surface tension (mN/m)	Density (g/cm^3)
1st	Non-treated	19.3	42.8	1.037
		18.9	39.0	1.045
		17.0	43.5	1.040
		-	41.7	-
	UV-irradiated	17.8	40.5	1.020

		16.7	38.4	1.048
		16.2	39.8	1.053
	Sonicated	5.25	24.1	0.999
		5.19	24.1	1.000
		5.07	26.3	1.050
60th	Non-treated	13.2	30.2	1.030
		13.8	31.1	1.050
		14.8	29.3	1.045
	UV-irradiated	14.1	28.4	1.043
		15.3	30.4	1.050
		13.2	29.0	1.058
	Sonicated	4.51	21.7	1.020
		4.56	23.1	0.997
		4.30	21.3	0.980

(A)

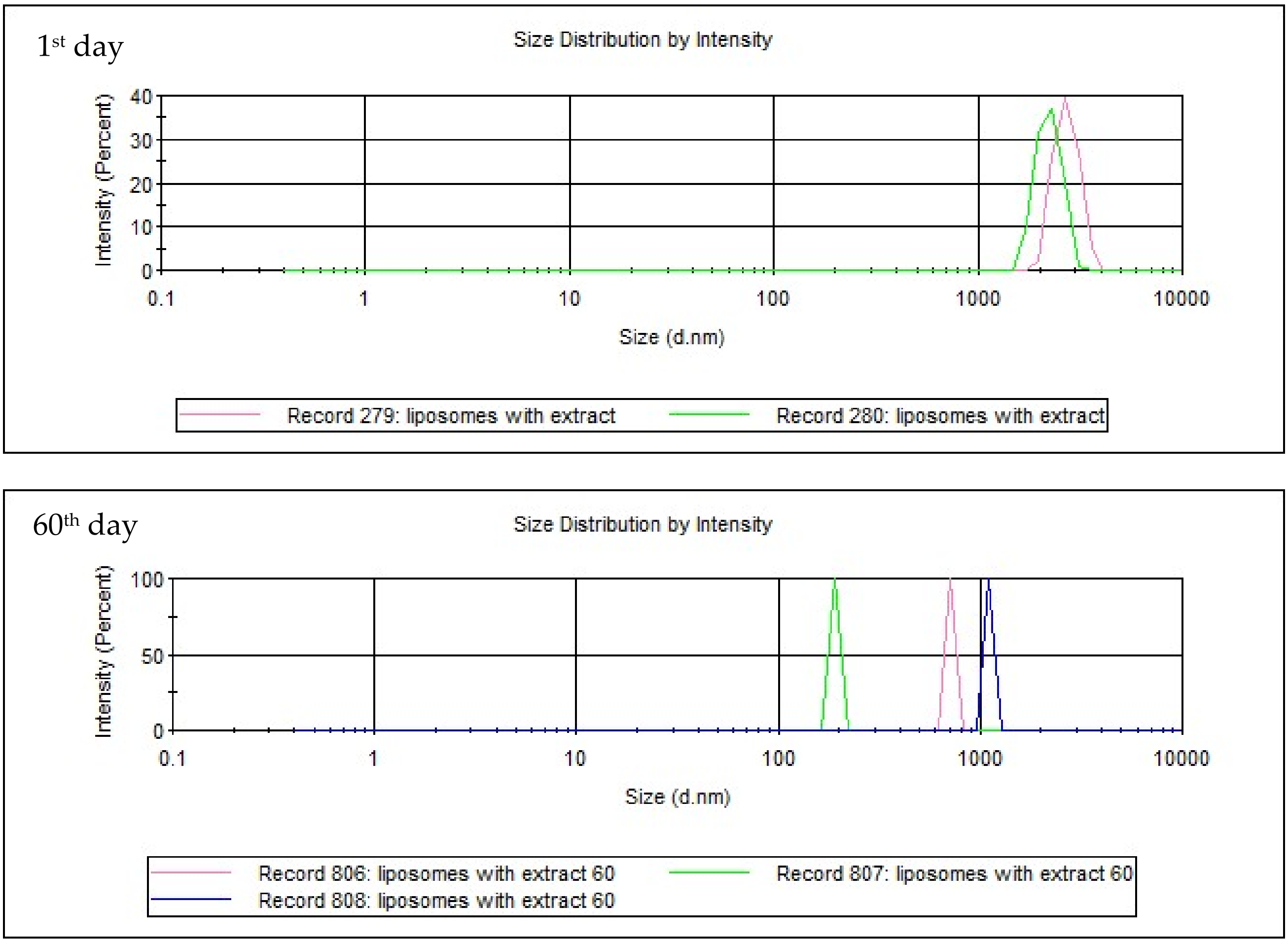

(B)

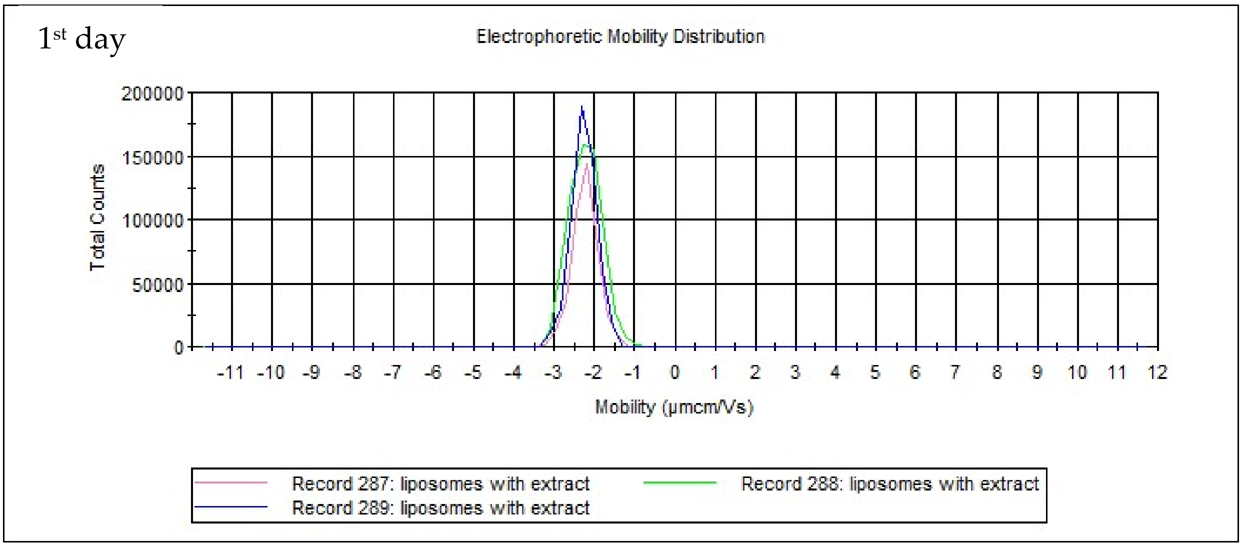
1st day
Electrophoretic Mobility Distribution
Total Counts
200000
150000
100000
50000
0
-11 -10 -9 -8 -7 -6 -5 -4 -3 -2 -1 0 1 2 3 4 5 6 7 8 9 10 11 12
Mobility (μmcm/Vs)
Record 287: liposomes with extract
Record 288: liposomes with extract
Record 289: liposomes with extract

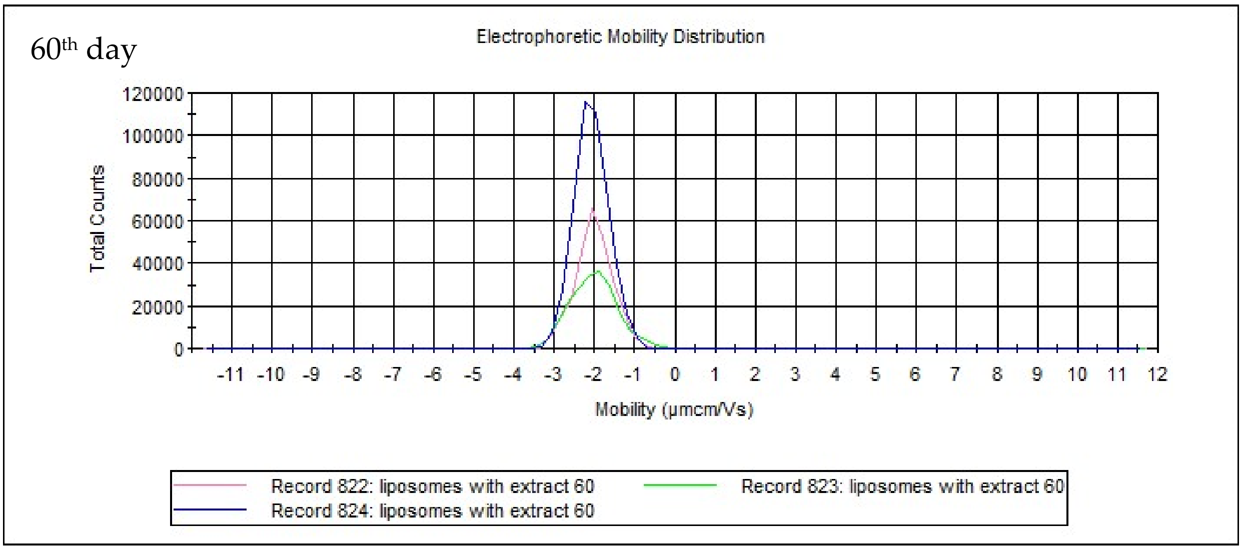
60th day
Electrophoretic Mobility Distribution
Total Counts
120000
100000
80000
60000
40000
20000
0
-11 -10 -9 -8 -7 -6 -5 -4 -3 -2 -1 0 1 2 3 4 5 6 7 8 9 10 11 12
Mobility (μmcm/Vs)
Record 822: liposomes with extract 60
Record 823: liposomes with extract 60
Record 824: liposomes with extract 60

(C)

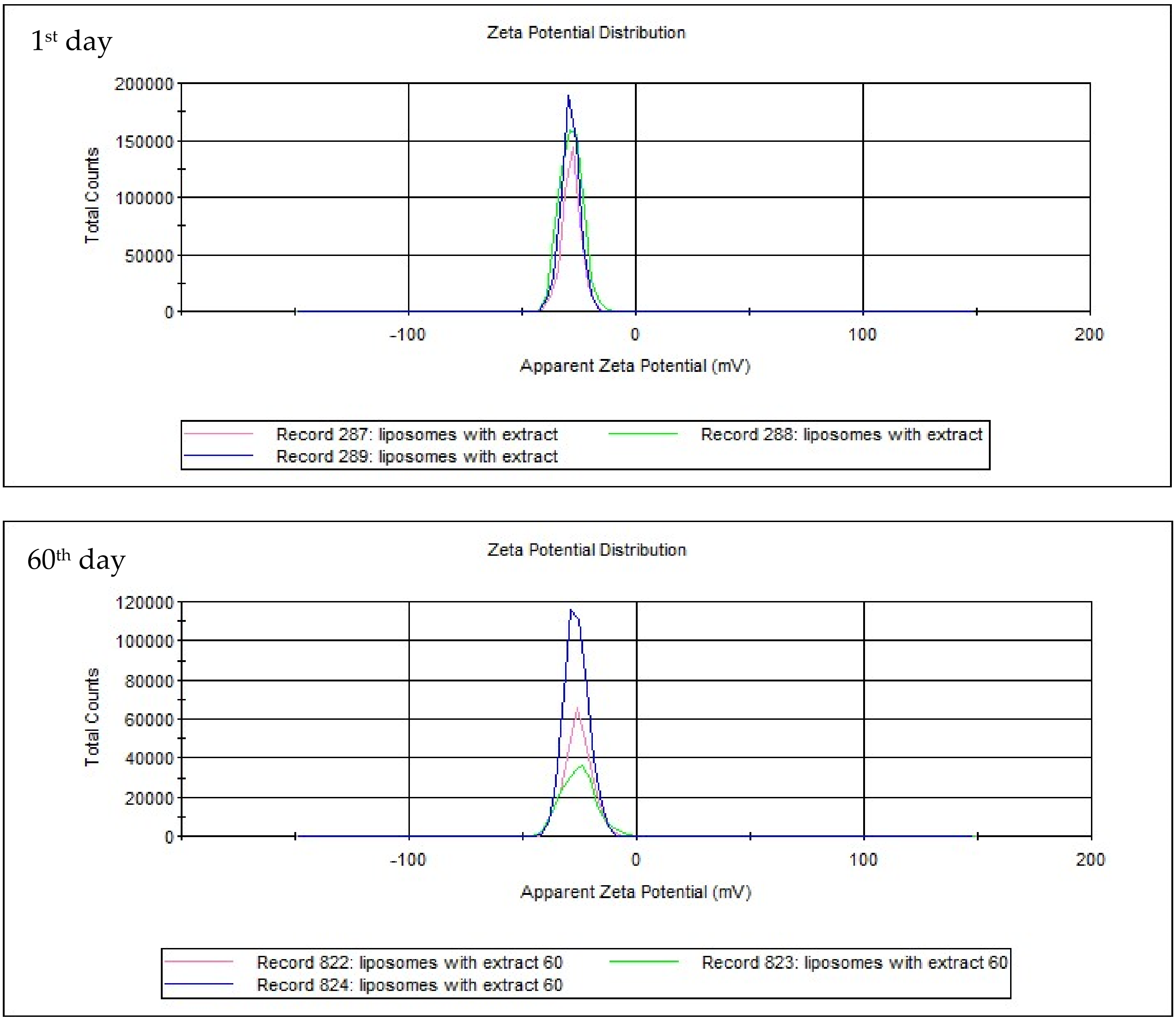

Figure S1. Graphical presentation of row data obtained using the dynamic light scattering method for non-treated carob extract-loaded liposomes at the 1st and the 60th days of storage at 4°C: (A) vesicle size, (B) mobility, and (C) zeta potential

(A)

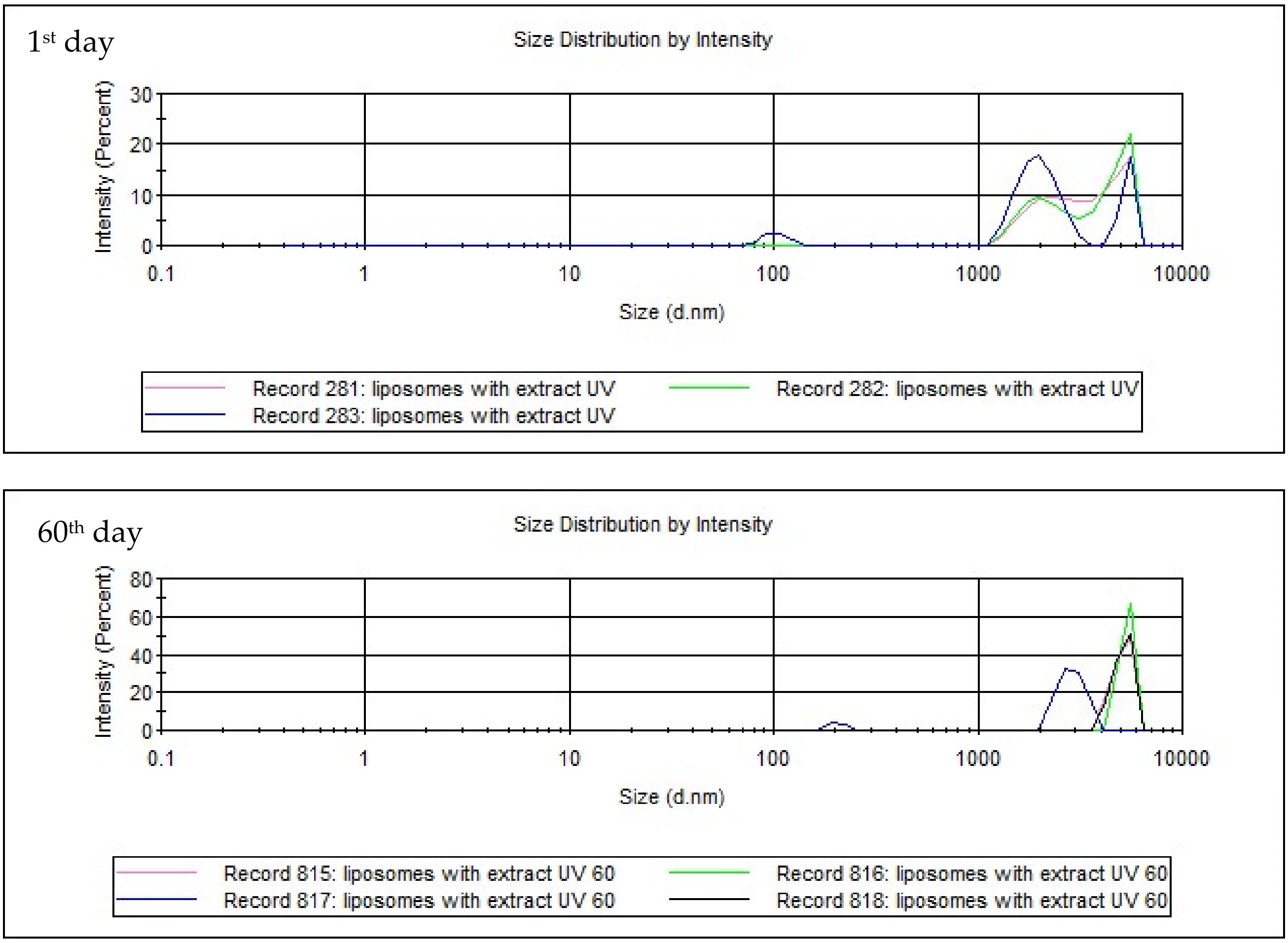
1st day
Size Distribution by Intensity
Intensity (Percent)
0
10
20
30
0.1
1
10
100
1000
10000
Size (d.nm)
Record 281: liposomes with extract UV
Record 282: liposomes with extract UV
Record 283: liposomes with extract UV
60th day
Size Distribution by Intensity
Intensity (Percent)
0
20
40
60
80
0.1
1
10
100
1000
10000
Size (d.nm)
Record 815: liposomes with extract UV 60
Record 816: liposomes with extract UV 60
Record 817: liposomes with extract UV 60
Record 818: liposomes with extract UV 60

(B)

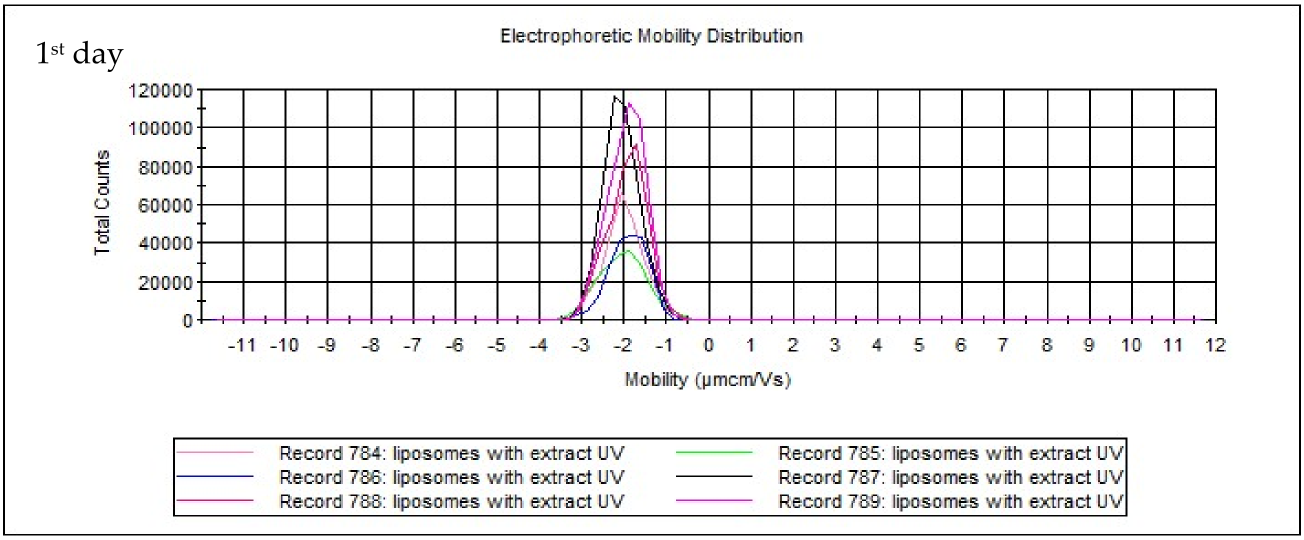
1st day
Electrophoretic Mobility Distribution
Total Counts
0
20000
40000
60000
80000
100000
120000
-11 -10 -9 -8 -7 -6 -5 -4 -3 -2 -1 0 1 2 3 4 5 6 7 8 9 10 11 12
Mobility (µmcm/Vs)
Record 784: liposomes with extract UV
Record 785: liposomes with extract UV
Record 786: liposomes with extract UV
Record 787: liposomes with extract UV
Record 788: liposomes with extract UV
Record 789: liposomes with extract UV

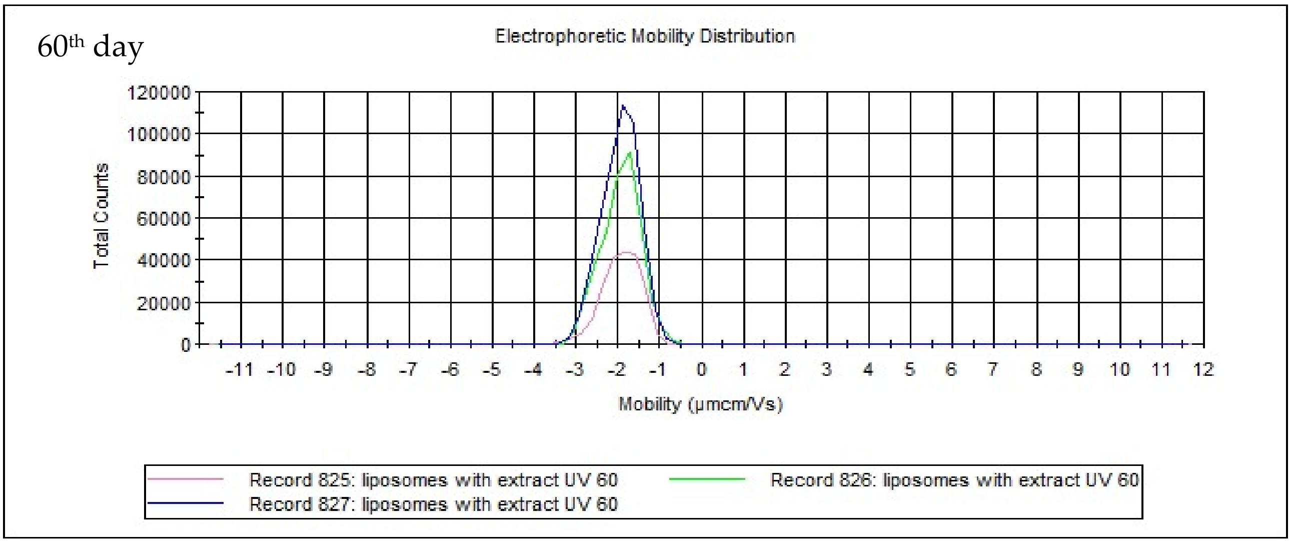

60th day
Electrophoretic Mobility Distribution
Total Counts
Mobility (μmcm/Vs)
Record 825: liposomes with extract UV 60
Record 826: liposomes with extract UV 60
Record 827: liposomes with extract UV 60

(C)

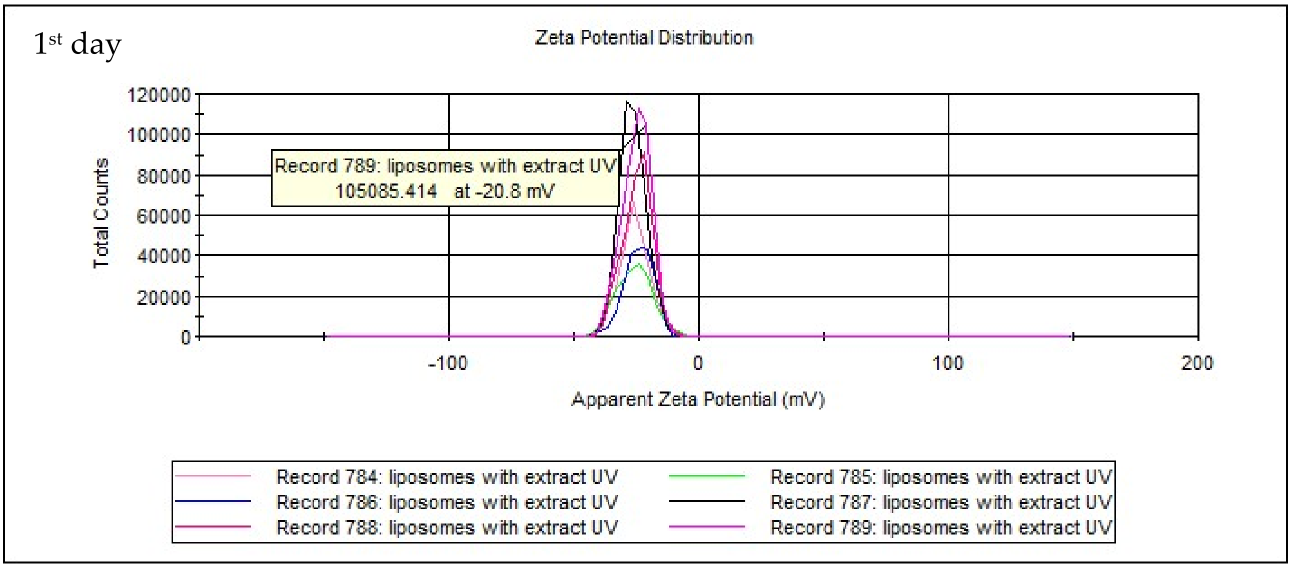

1st day
Zeta Potential Distribution
Record 789: liposomes with extract UV
105085.414 at -20.8 mV
Total Counts
Apparent Zeta Potential (mV)
Record 784: liposomes with extract UV
Record 785: liposomes with extract UV
Record 786: liposomes with extract UV
Record 787: liposomes with extract UV
Record 788: liposomes with extract UV
Record 789: liposomes with extract UV

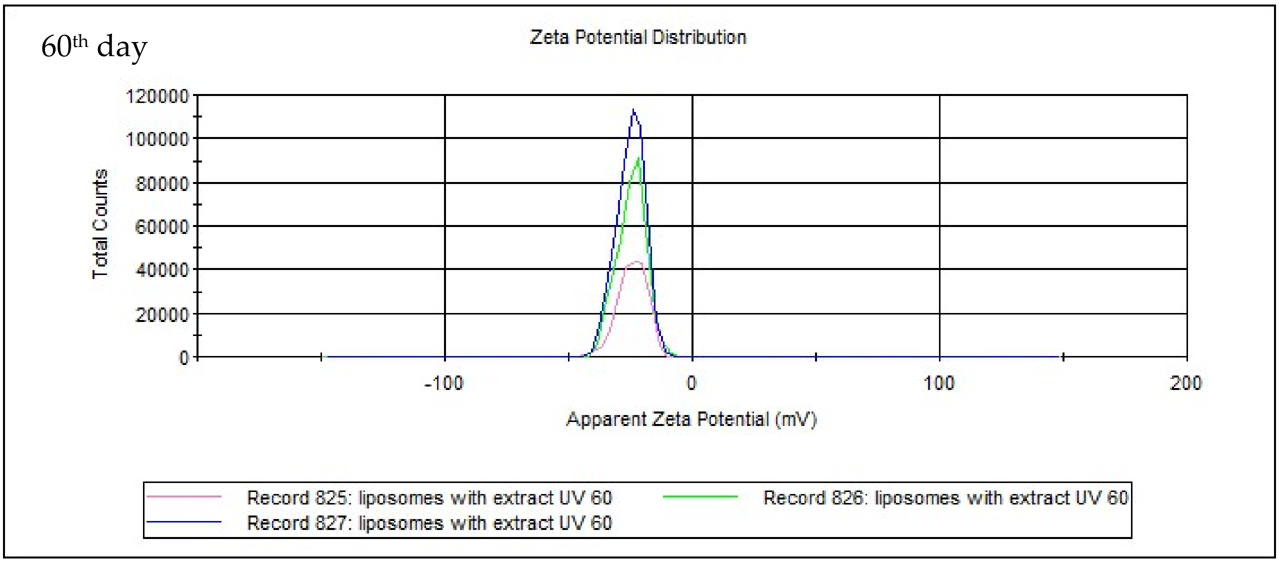

Figure S2. Graphical presentation of row data obtained using the dynamic light scattering method for UV-irradiated carob extract-loaded liposomes at the 1st and the 60th days of storage at 4°C: (A) vesicle size, (B) mobility, and (C) zeta potential

(A)

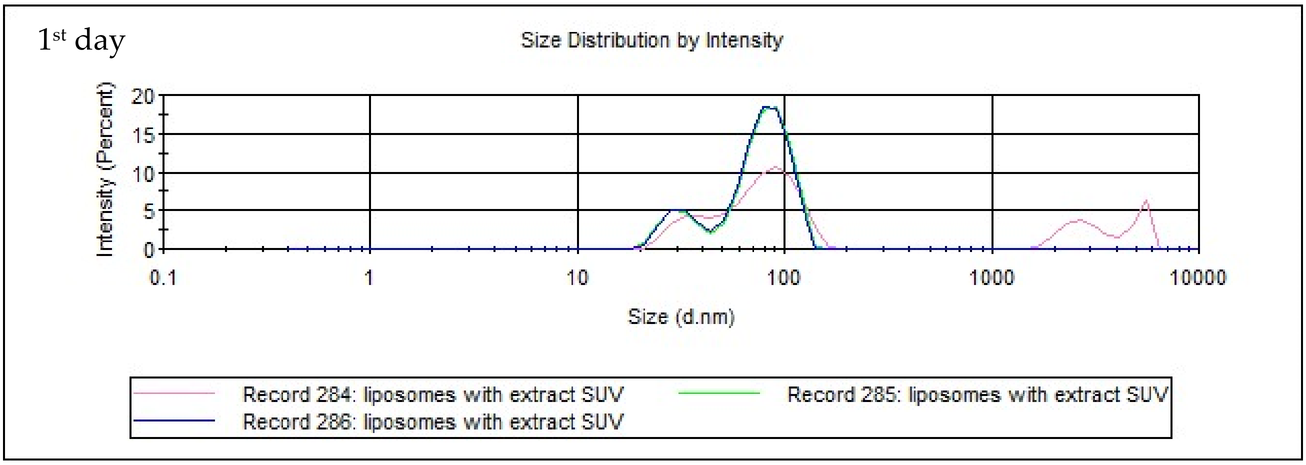

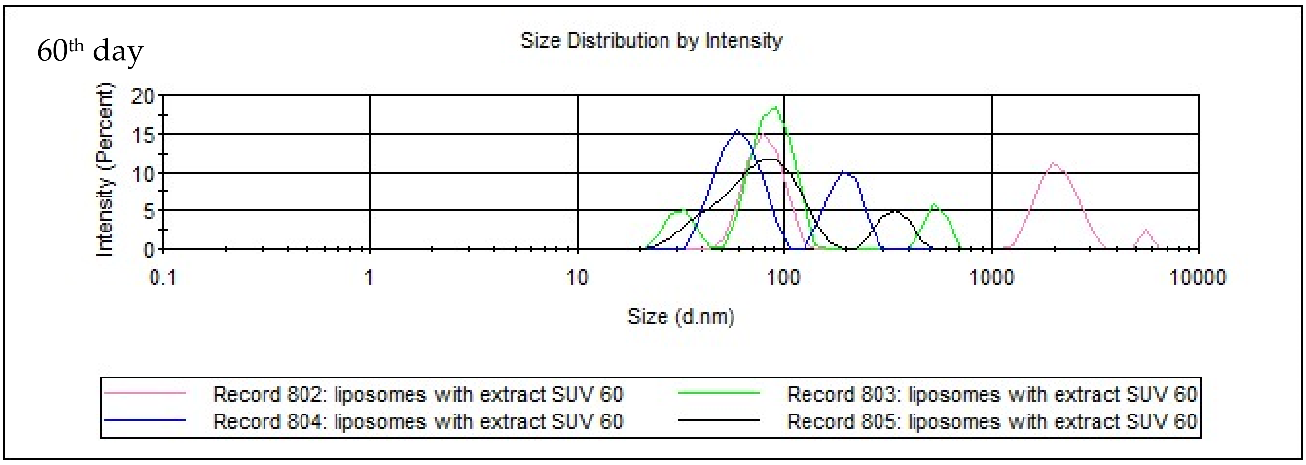
60th day
Size Distribution by Intensity
Intensity (Percent)
0
5
10
15
20
0.1
1
10
100
1000
10000
Size (d.nm)
Record 802: liposomes with extract SUV 60
Record 803: liposomes with extract SUV 60
Record 804: liposomes with extract SUV 60
Record 805: liposomes with extract SUV 60

(B)

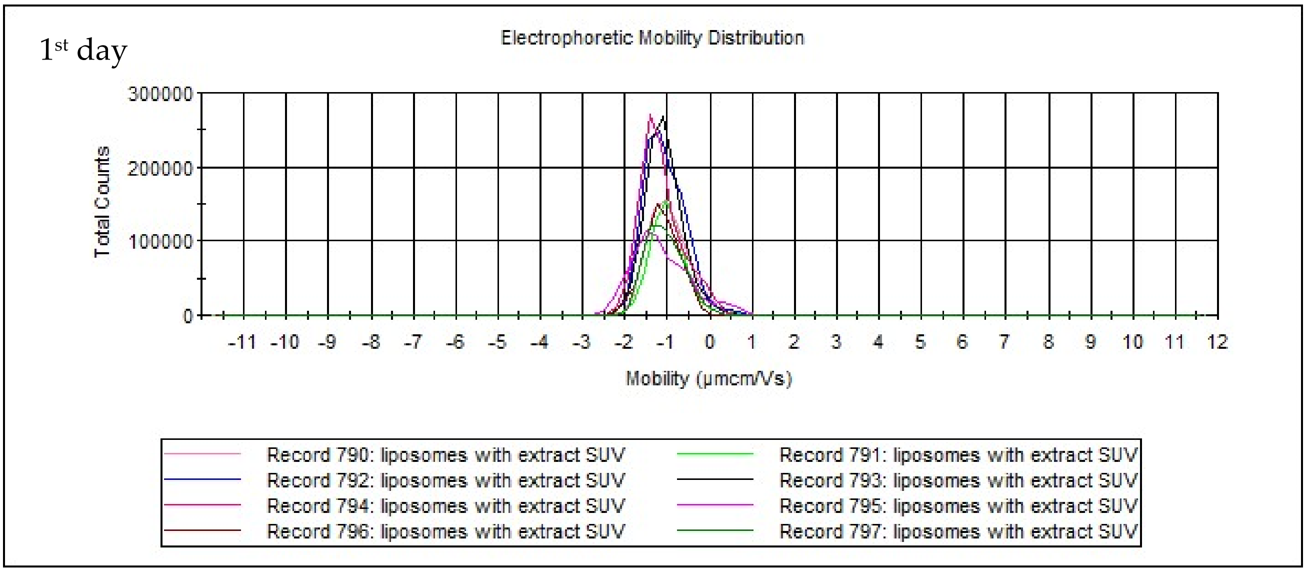
1st day
Electrophoretic Mobility Distribution
Total Counts
0
100000
200000
300000
-11 -10 -9 -8 -7 -6 -5 -4 -3 -2 -1 0 1 2 3 4 5 6 7 8 9 10 11 12
Mobility (μmcm/Vs)
Record 790: liposomes with extract SUV
Record 791: liposomes with extract SUV
Record 792: liposomes with extract SUV
Record 793: liposomes with extract SUV
Record 794: liposomes with extract SUV
Record 795: liposomes with extract SUV
Record 796: liposomes with extract SUV
Record 797: liposomes with extract SUV

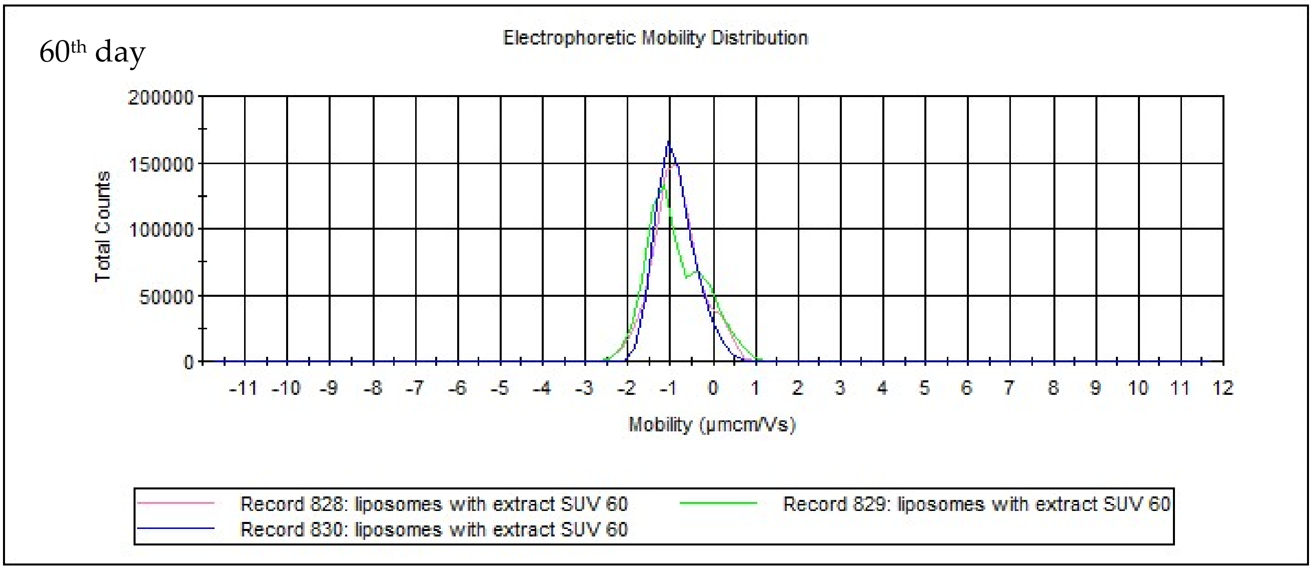
60th day
Electrophoretic Mobility Distribution
Total Counts
0
50000
100000
150000
200000
-11 -10 -9 -8 -7 -6 -5 -4 -3 -2 -1 0 1 2 3 4 5 6 7 8 9 10 11 12
Mobility (μmcm/Vs)
Record 828: liposomes with extract SUV 60
Record 829: liposomes with extract SUV 60
Record 830: liposomes with extract SUV 60

(C)

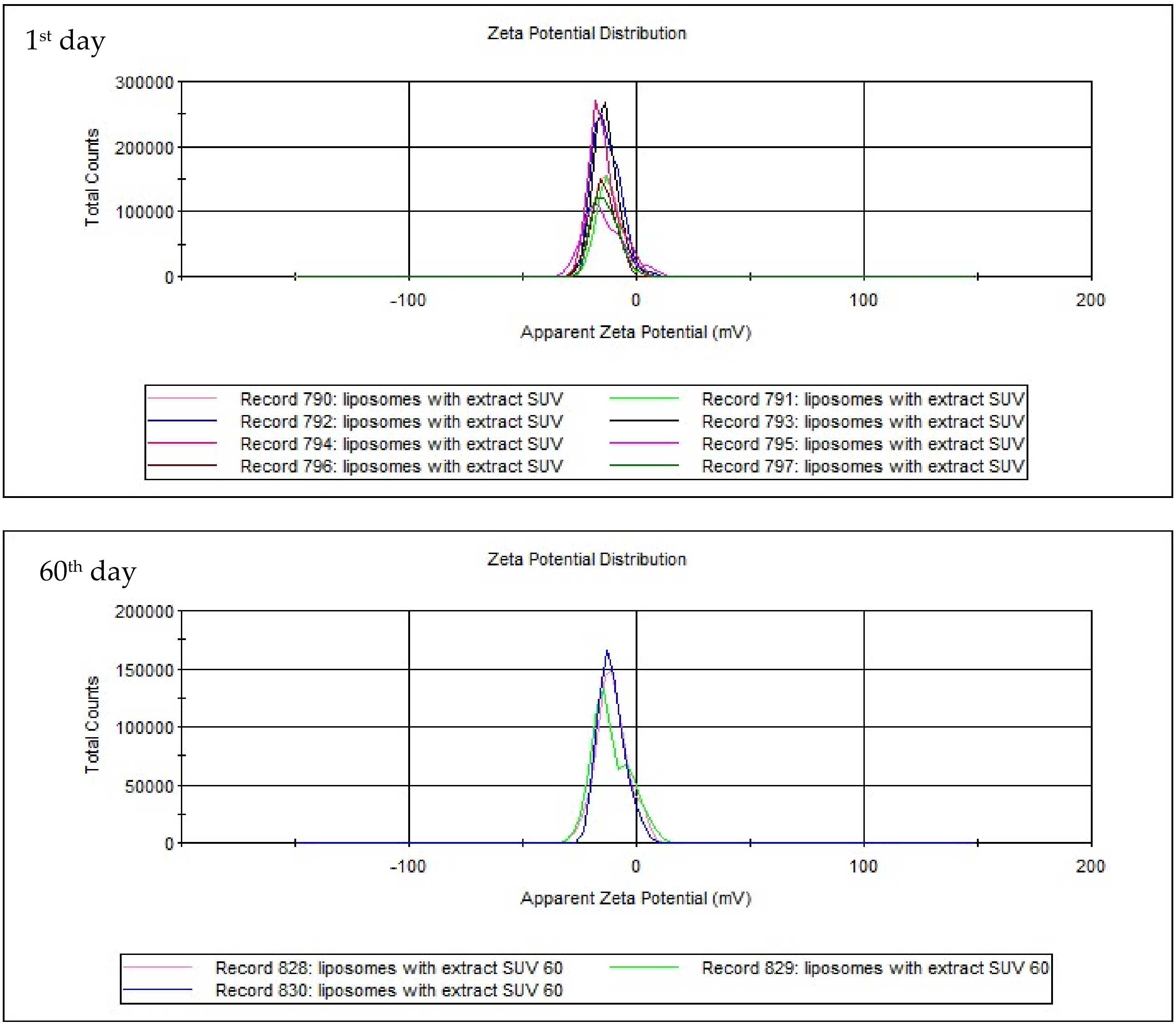

Figure S3. Graphical presentation of row data obtained using the dynamic light scattering method for sonicated carob extract-loaded liposomes (SUV) at the 1st and the 60th days of storage at 4°C: (A) vesicle size, (B) mobility, and (C) zeta potential

(A)

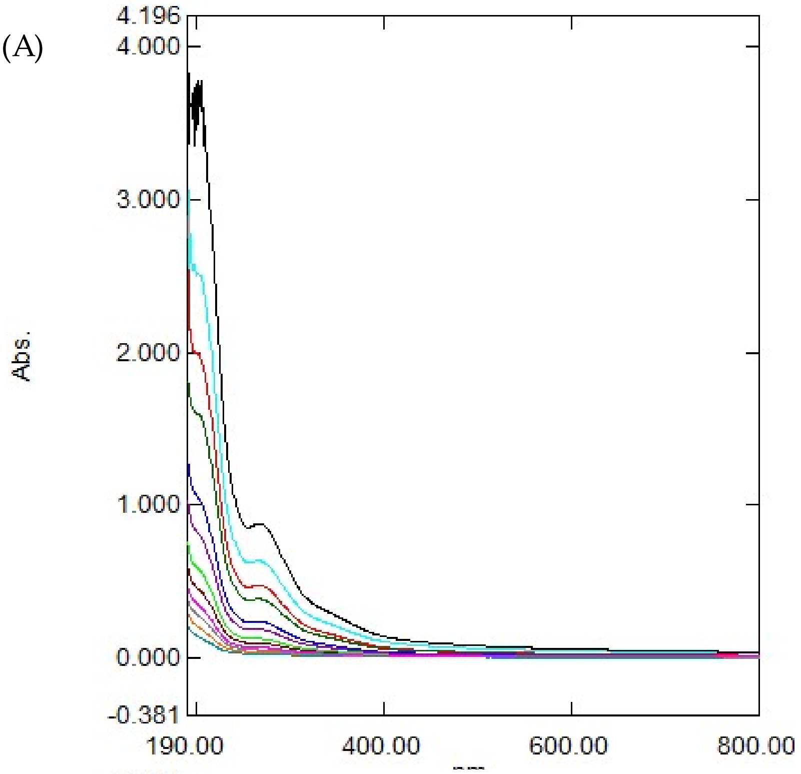
4.196
4.000
3.000
2.000
1.000
0.000
-0.381
Abs.
190.00
400.00
600.00
800.00

(B)

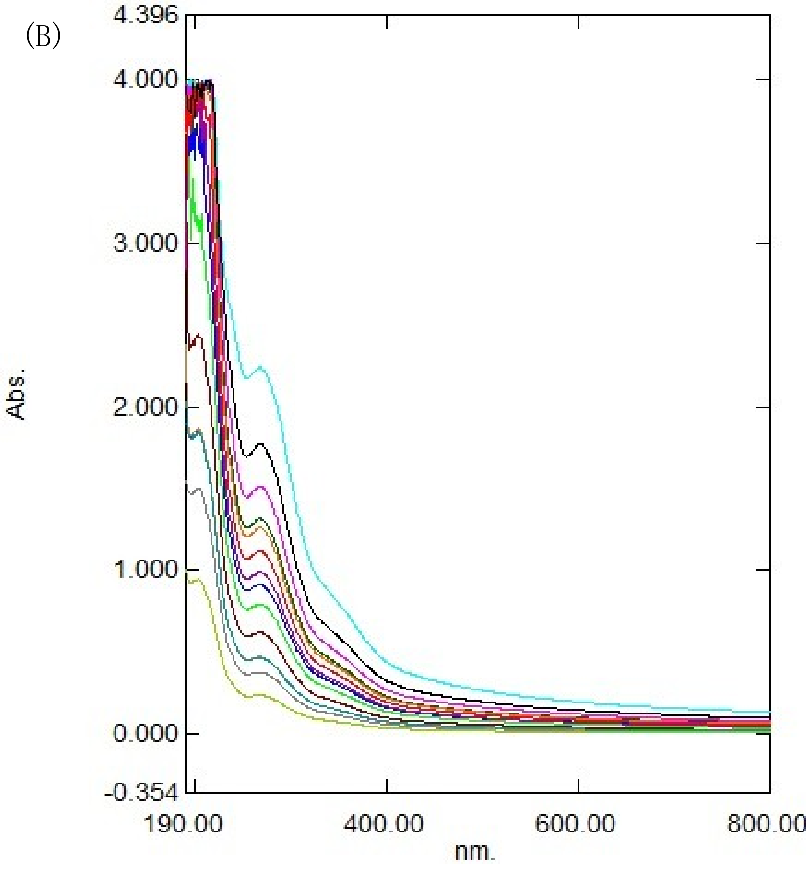
4.396
4.000
3.000
2.000
1.000
0.000
-0.354
Abs.
190.00
400.00
600.00
800.00
nm.

Figure S4. UV spectra of carob extract polyphenols in (A) simulated gastric fluid (SGF, pH 1.5) and (B) simulated intestinal fluid (SIF, pH 7.4)
